# To Subscribers

**Published:** 1858-12

**Authors:** 


### EDITORIAL AND MISCELLANEOUS.

### TO SUBSCRIBERS.

Quite a large number of subscribers who were in arrears, have responded promptly to bills which we sent out some weeks ago, it is hoped that others will do likewise before long, as there still stands upon our books a very large amount of indebtedness.

We have good reason to believe, from the assurances which have reached us, that the journal we are furnishing, far exceeds in value the mere trifle which we charge in the way of subscription.

We agree fully with the New Orleans Medical Gazette "that Medical Journals are furnished far below a remunerating price," and contend that those who furnish a large number of practitioners of medicine with perhaps the whole of their medical reading, and the only means they have or will avail themselves of for keeping pace with the progress of the science, should do this without an adequate remuneration, is a reproach to the profession.

It may be said by some that there are too many Medical Journals, and if there were a smaller number they would be better supported, but in reply to this we would say, that it is a notorious fact that there are hundreds and perhaps thousands of Medical men in the United States who do not take even a single Medical Journal, and if they could be aroused to a sense of their own interests and what is due to their calling and the responsible positions they occupy, every journal in the land would not only be sustained, but yield a handsome remuneration to those who are now, in many instances, receiving no compensation whatever for the arduous labors connected with the position of a Medical Editor.

We have occasionally received intimations of mistakes that are supposed to have been made in the accounts sent out, in reply to which, all we can say is, that we shall leave disputed questions of this character to be settled by the honor of those who receive the journal, not intending however to hold any further communication with those who prove themselves to be unworthy of confidence. Some *real* mistakes doubtless occur, and it will at all times afford us great pleasure to correct them, and we therefore earnestly solicit notifications of the fact where errors have been committed in the accounts sent out.

We still want an additional 1000 subscribers of the right stamp; those who conform to our terms and appreciate our labors—not those who consider it an honor conferred upon us, to *allow* the Journal to be sent to their address—we have never felt disposed to acknowledge it an honor, as some people seem to conceive it, to be permitted to render our professional services gratuitously, and we attach as little importance, in an editorial capacity, to the patronage of those who do not estimate our services as of sufficient value, to *desire* them, and be willing to make a prompt and adequate return.

We do, however, feel grateful to the many recipients of our Journal labors, who have continually manifested their appreciation of our efforts, not only in a pecuniary way (valued mainly, because indispensable to our very existence) but by kind words and encouraging assurances have given us strength and perseverance in a pursuit, which has in a money sense, been largely unrewarded.

Our Journal is in its fourth volume, and may now be looked upon as a permanent "Institution," and we promise those who feel an interest in its progressive success, if they will only furnish us the money,—through a sufficient number of

*paying,* subcribers—to *command* an amount of science and talent, which will in return, give them a Journal not inferior to any in the United States. This is the object of our ambition. Who will contribute to its realization?

